# How Undergraduates Perceive Stress Factors: An exploratory study

**DOI:** 10.12669/pjms.39.4.7760

**Published:** 2023

**Authors:** Manabu Murakami, Akiko Takeuchi, Shigeki Jin, Kotaro Matoba

**Affiliations:** 1 Manabu Murakami, MD, PhD. Center for Medical Education and International Relations, Faculty of Medicine, Hokkaido University, Kita 15, Nishi 7, Kita-ku, Sapporo, Hokkaido 060-8638, Japan; 2 Akiko Takeuchi, DDS, PhD. Department of Forensic Medicine, Faculty of Medicine, Hokkaido University, Kita 15, Nishi 7, Kita-ku, Sapporo, Hokkaido 060-8638, Japan; 3 Shigeki Jin, PhD. Department of Forensic Medicine, Faculty of Medicine, Hokkaido University, Kita 15, Nishi 7, Kita-ku, Sapporo, Hokkaido 060-8638, Japan; 4 Kotaro Matoba, MD, PhD. Department of Forensic Medicine, Faculty of Medicine, Hokkaido University, Kita 15, Nishi 7, Kita-ku, Sapporo, Hokkaido 060-8638, Japan

Saeed et al.’s 2021 study examined the impact of Coronavirus disease (COVID-19) on anxiety and depression among students.^1^ Our team was interested in the mental health of undergraduate students and intended to investigate the stressors affecting students’ mental health even prior to the COVID-19 crisis. Weber et al.’s pre-COVID-19 report found that factors such as examination, poor teaching, heavy workload, time pressure, and financial hardship affected student’s mental health.^2^ We predicted that COVID-19 added new stressors such as social disconnection, and clarified these stressors through interviews.

We interviewed 34 first-to third-year undergraduate Japanese students, and asked them “what are the factors that made you feel stressed in your studies or private school life?” Students’ interviews were recorded and transcribed. The transcribed data was coded, and similar contents were classified into five mutually exclusive categories with qualitative content analysis as follows: 1. Transition to university life; 2. Examinations; 3. Attendance and homework; 4. Extracurricular activities; 5. Social and human relationships (Table-I). COVID-19-specific questions were included in all categories, and in particular, many comments were found in categories 3, 4, and 5, which coincided with the results of a previous study by Ansari et al.^3^

**Table-I:** Brief Summary of student interviews regarding stress factors.

Categories	Explanation of the Categories	Examples of Students’ Comments
1. Transition to university life	Feeling the stress that they did not experience in high school because of living alone and away from their parents and working part-time to make a living.	*“I live alone, and my health is getting worse. If I was at my parent’s house, my parents would take care of me and serve me meals, but now that I live alone, I have to cook my own meals and have to do my personal care completely by myself.”*
2. Examinations	Feeling stress because of having to memorize considerable knowledge in a short period of time to pass the exams and complete credits.	*“There are constant tests all the time. I am always conscious of the tests. I have another test next week, so I have to memorize a lot every day. So, my life is regulated, and I feel very stressful.”*
3. Attendance and homework	Feeling stress because of an overloaded schedule of lectures and practices, especially the impact of being assigned homework in multiple subjects.	*“I have extreme anxiety… (Due to the pandemic). I have no face-to-face classes and attendance is supposed to be checked with submitting the assigned homework. I think about my homework all the time even when I’m taking a bath or going to bed, even on my days off. I can’t focus on enjoying my hobbies even on Saturdays or Sundays.”*
4. Extracurricular activities	Feeling stress owing to not being able to participate in club activities, including sports or cultural activities and having to stay at home because of the pandemic.	*“I cannot go far away to eat or see something due to the pandemic. I’m just not dead! I haven’t been able to do any activities that can feel fun as a university student. I’m waiting time…”*
5. Social and human relationships	Feeling stress owing to social distancing, which weakens face-to-face communications with family, friends, and faculty. This includes the disruption of life rhythms owing to the lack of commuting to campus.	*“I don’t even know who to talk to because I meet my friends only through SNS. I’d like to meet my friends face-to-face and talk to them, but I can’t really do that. If I could meet them in person, I’d like to talk with my friends about their past lives or things like that.”*

Ansari et al. (2022) published data on stress and anxiety in medical and non-medical students; using a quantitative cross-sectional online survey, they found that the effect of social distancing and online learning weakened the students’ relationships with their family, their peers, and the faculty,^3^ which is consistent with the findings of our qualitative research. While the methods used in Ansari et al.’s study and the present study differed, the commonality in these findings regarding newly emerging factors could be considered a global indicator of risk factors in students’ mental health.

We strongly agree with the conclusion of Ansari et al.’s study that social connection/support should be maintained as much as possible even in a virtual setting^3^ for students to sustain positive mental health. Moreover, as suggested by Saeed et al., there is an urgent need for stress management, improvement of unhealthy lifestyle, and referral to appropriate psychiatrists^1^ for mental health with post COVID-19 medical education.

## Authors’ Contributions:

**MM:** Research concept, design of the study, literature review, data collection, data analysis, data interpretation, preparation of initial draft, approval of final draft, and guarantor of the manuscript.

**SJ:** Research concept, design of the study, literature review, data collection, data analysis, data interpretation, revision of initial draft, approval of final draft, and corresponding author of the manuscript.

**AT:** Research concept, design of the study, literature review, data interpretation, revision of initial draft, and approval of final draft.

**KM:** Research concept, design of the study, prior literature review, data interpretation, revision of initial draft, and approval of final draft.
